# Study of the Possibility of Using Sol–Gel Technology to Obtain Magnetic Nanoparticles Based on Transition Metal Ferrites

**DOI:** 10.3390/gels9030217

**Published:** 2023-03-14

**Authors:** Nina Shabelskaya, Sergey Sulima, Elena Sulima, Oleg Medennikov, Marina Kulikova, Tatyana Kolesnikova, Svetlana Sushkova

**Affiliations:** 1 Department of Ecology and Industrial Safety, Faculty of Technology, Platov South-Russian State Polytechnic University (NPI), 346400 Novocherkassk, Russia; 2 Laboratory “Agrobiotechnologies for Improving Soil Fertility and Quality of Agricultural Products”, D.I. Ivanovsky Academy of Biology and Biotechnology, Southern Federal University, 344090 Rostov-on-Don, Russia; 3 Department of Chemical Technologies, Faculty of Technology, Platov South-Russian State Polytechnic University (NPI), 346400 Novocherkassk, Russia

**Keywords:** sol–gel method, cobalt (II) ferrite, composite materials, magnetic nanoparticles, magnetic composites

## Abstract

The article presents results for the magnetic nanoparticles sol–gel method synthesis of cobalt (II) ferrite and organic–inorganic composite materials based on it. The obtained materials were characterized using X-ray phase analysis, scanning and transmission electron microscopy, Scherrer, Brunauer–Emmett–Teller (BET) methods. A composite materials formation mechanism is proposed, which includes a gelation stage where transition element cation chelate complexes react with citric acid and subsequently decompose under heating. The fundamental possibility of obtaining an organo–inorganic composite material based on cobalt (II) ferrite and an organic carrier using the presented method has been proved. Composite materials formation is established to lead to a significant (5–9 times) increase in the sample surface area. Materials with a developed surface are formed: the surface area measured by the BET method is 83–143 m^2^/g. The resulting composite materials have sufficient magnetic properties to be mobile in a magnetic field. Consequently, wide possibilities for polyfunctional materials synthesis open up for various applications in medicine.

## 1. Introduction

An increase in the technogenic load on an individual in modern society leads to the development of many diseases, including cancer. Research in the field of medicine is becoming more and more interdisciplinary, new methods of treatment are created. Interventional procedures are widely used [1,2,3], such as transarterial chemoembolization, catheter intra-arterial delivery of nanoparticles, etc. The possibilities of non-surgical and drug-free direct selective influence on individual cells in an external magnetic field by oscillations of magnetic nanoparticles fixed on the cell membranes or introduced into cells have been investigated [4,5,6,7]. Study of the processes of synthesis of organo–inorganic composites is an interesting and challenging task. The formation of dual-use materials containing an inorganic magnetic part and an organic one will expand the functional capabilities of substances. Ferrites transition elements are promising candidates for medical applications due to a successful combination of their technical parameters: ease of synthesis and presence of magnetic properties. Iron oxide compounds exhibit antibacterial properties and are nontoxic [8], which allows them to be considered as promising materials for theranostics [9,10]. The nanoparticles’ developed surface displays an increased adsorption activity [11], which can be used for organic–inorganic composite materials synthesis [12,13]. One of the promising methods for obtaining magnetic nanoparticles is the sol–gel method [14,15,16]. Often these processes are lengthy and time-consuming. For example, in [16], transition metal salts were dissolved in deionized water, citric acid was added, and stirred for 1 h. The mixed solution was heated in a water bath at 95 °C for 3 h. Then, the resulting transparent sol was dried at a temperature of 95 °C for 12 h. Finally, the mixture was calcined at 400 °C for 2 h in a muffle furnace. Therefore, the main goal of the study is to investigate the possibility of using simply the sol–gel technology to obtain cobalt (II) ferrite magnetic nanoparticles and composite organo–inorganic materials based on them.

## 2. Results and Discussion

During the synthesis of cobalt (II) ferrite, when solutions of transition metal nitrates and ammonia were mixed, a brown amorphous precipitate formation was observed, which may be associated with the preparation of a mixture of cobalt (II) and iron (II) hydroxides according to Reactions (1) and (2):

Co(NO_3_)_2_ + 2NH_4_OH = Co(OH)_2_ + 2NH_4_NO_3_ (1)

Fe(NO_3_)_3_ + 3NH_4_OH = Fe(OH)_3_ + 3NH_4_NO_3_ (2)

with the introduction of a citric acid solution, the precipitate dissolved. This may be due to the transition metal citrates formation according to Reactions (3) and (4):

Co(OH)_2_ + C_6_H_8_O_7_ = Co(C_6_H_6_O_7_) + 2H_2_O (3)

Fe(OH)_3_ + C_6_H_8_O_7_ = Fe(C_6_H_5_O_7_) + 3H_2_O, (4)

During further heat treatment, the solution first turned into a viscous gel and then into a solid porous material. Presumably, bulky citrate complexes of transition metals were formed. In this case, citric acid acted in a dual role: as an organic fuel and a dispersing agent that prevented the formation of cobalt (II) ferrite large particles. When the heat treatment stopped, the formation of a black powder was observed.

**Figure 1:**
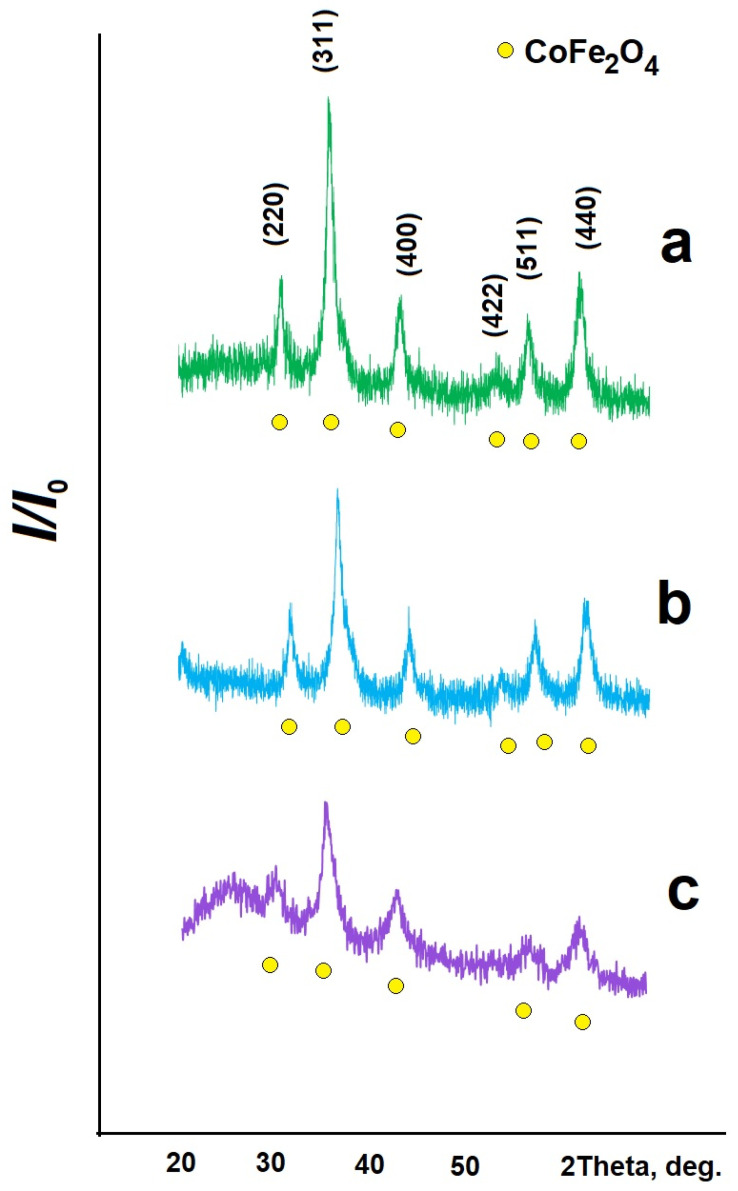
X-ray pattern of synthesized samples: (**a**)—CoFe_2_O_4_, (**b**)—CoFe_2_O_4_/C, (**c**)—CoFe_2_O_4_/S.

Figure 1a shows an FC sample X-ray diffraction pattern. Analysis of the X-ray diffraction pattern reveals that the sample is only cobalt (II) ferrite having a cubic spinel structure (PDF Number 010-74-6403). The array parameter *a* and the crystallite sizes *D* calculated with the Scherrer formula are given in Table 1. The lines of the diffractogram for pure cobalt (II) ferrite are the clearest and the most pronounced in comparison with similar lines for other synthesized materials (FC/C and FC/S composites). This may be due to the formation of the most defect-free sample.

Figure 1b,c show X-ray diffraction patterns of the synthesized composite materials. They are represented by lines characteristic of cobalt (II) ferrite (PDF Number 010-74-3419 for FC/C (Figure 1b), PDF Number 010-74-6403 for FC/S (Figure 1c)) (the carbon part is X-ray amorphous).

Remarkably, cobalt (II) ferrite formation on the surface of an organic support leads to the formation of smaller magnetic particles, as the diffraction pattern peaks are blurred and less clear.

**Figure 2:**
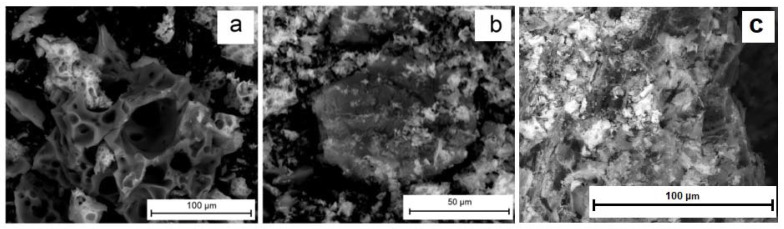
Micrographs of synthesized samples: (**a**)—CoFe_2_O_4_, (**b**)—CoFe_2_O_4_/C, (**c**)—CoFe_2_O_4_/S.

**Figure 3:**
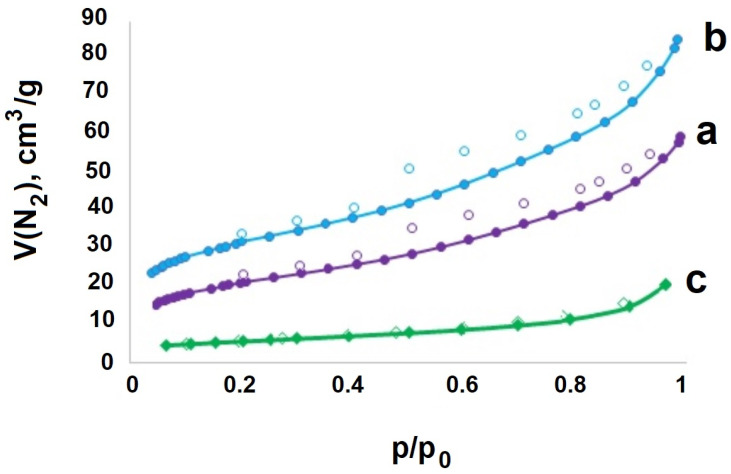
Adsorption–desorption isotherms of N_2_ samples: (**a**)—CoFe_2_O_4_/S, (**b**)—CoFe_2_O_4_/C, (**c**)—CoFe_2_O_4_.

Figure 2a, shows an FC sample micrograph (SEM image). Evidently, a porous material is formed. The specific surface area values (*S*_BET_) measured by the BET method are given in Table 1. Figure 3 shows the adsorption–desorption isotherms of N_2_.

**Figure 4:**
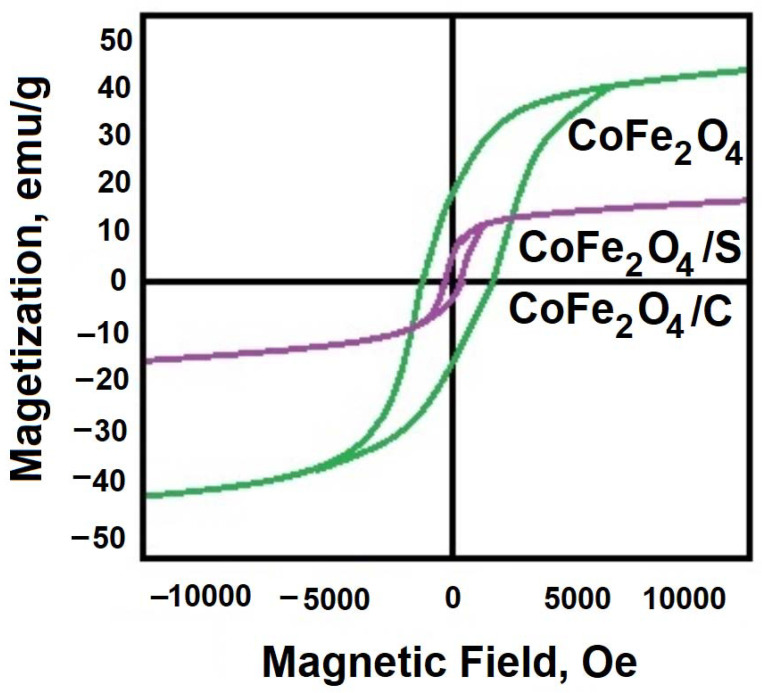
Magnetic hysteresis loops of synthesized samples.

The resulting cobalt (II) ferrite exhibits the properties of a magnetic material. Figure 4 shows the magnetic hysteresis loops of synthesized samples. According to the measurements, it demonstrates saturation magnetization of *M*_S_ = 44 emu/g (electromagnetic unit/g).

For a possible use as a targeted medication delivery carrier, for example, composite materials with a magnetic and an organic components formation by the sol–gel method were necessary to study. For this purpose, the FC/S and FC/C composite material synthesis was carried out. In the process, all technological operations were carried out similarly to those described above, with one difference: either biochar from sunflower husks or activated carbon from coconut shell, respectively, were initially placed in the reaction vessel.

As a result, an organic–inorganic composite material was successfully obtained, consisting of cobalt (II) ferrite and a carbon-containing component.

**Figure 5:**
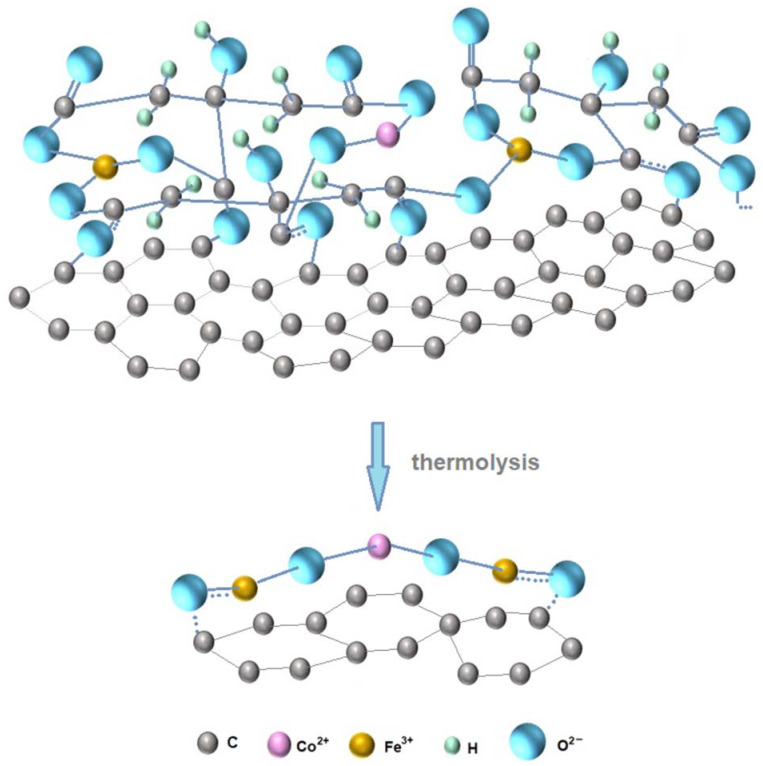
Possible structure of the intermediate citrate complex of transition elements.

Figure 5 shows a schematic representation of citrate complexes formation and cobalt (II) ferrite formation on the surface of a carbonaceous material.

**Figure 6:**
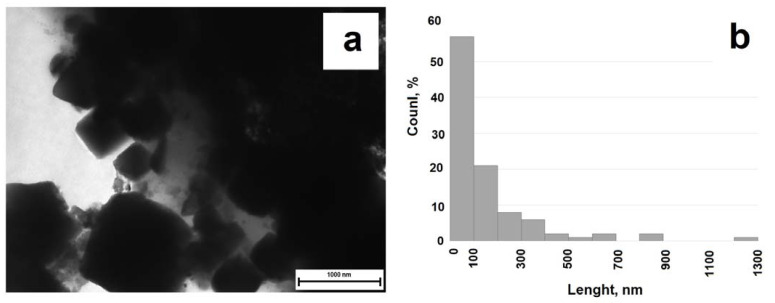
TEM image of the sunflower/CoFe_2_O_4_ biochar sample (**a**), particle size distribution (**b**).

Figure 2b shows an FC/C composite material sample micrograph. Cobalt (II) ferrite cluster formation on the surface of a carbon particle is observed. A similar effect is noted for the FC/S composite formation; see Figure 6a for a TEM image of the synthesized material. Regular geometric cobalt (II) ferrite particles in the shape of octahedrons are observed to form on the biochar surface. The particle sizes are shown in Figure 6b. The particle size of cobalt (II) ferrite lies in the range up to −200 nm. The crystallite sizes calculated by the Scherrer method, and the surface area values measured by the BET method, are presented in Table 1.

**Figure 7:**
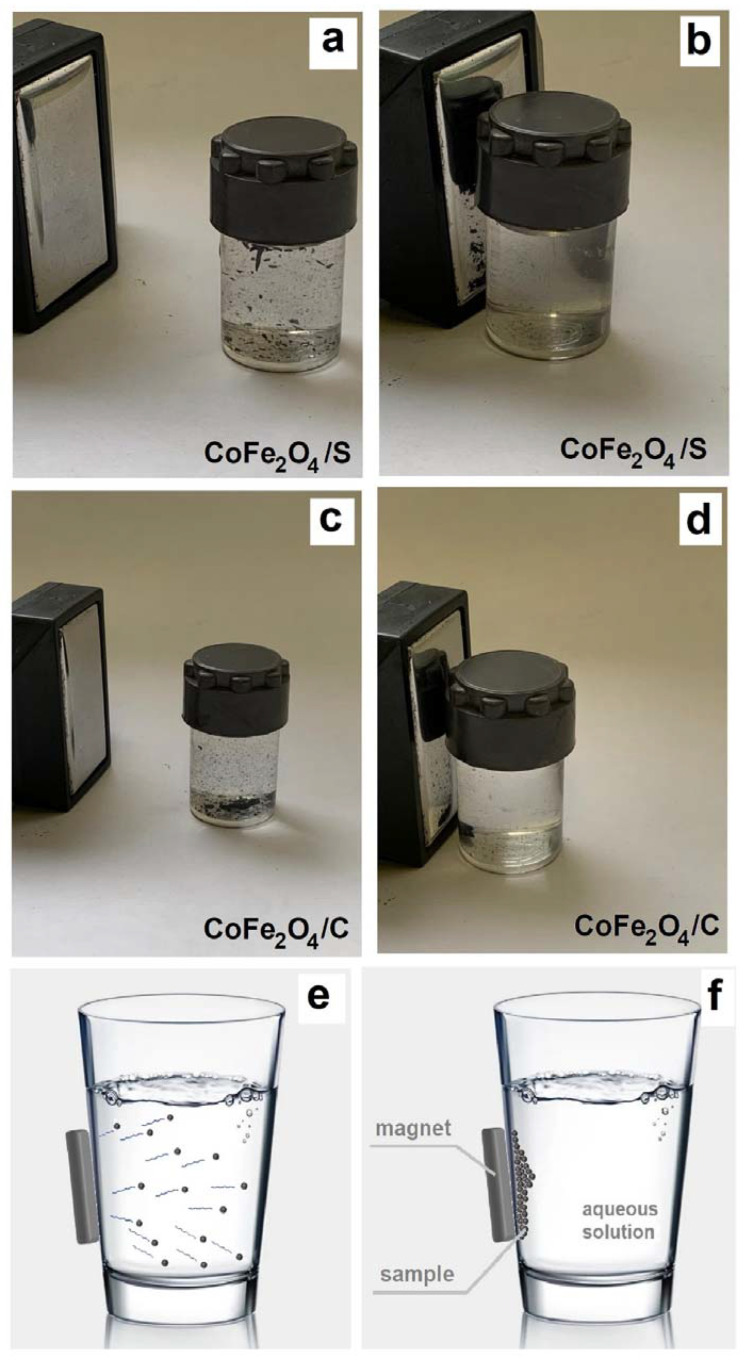
Magnetic separation of synthesized material: composites (**a**–**d**), schematic representation (**e**,**f**).

The organic–inorganic composite materials formation is accompanied by a significant (5–9 times) increase in the surface area of the material (see Table 1). This result suggests a higher activity of the synthesized materials in comparison with pure cobalt (II) ferrite in the processes associated with surface activity—adsorption, catalytic. At the same time, the synthesized composites display sufficiently pronounced magnetic characteristics (with a value of *M*_S_ = 14 emu./g for CoFe_2_O_4_/S (and CoFe_2_O_4_/C)) to be moved in an aqueous solution by a magnet (see Figure 7).

The synthesized materials were tested in the process of adsorption of chromium (VI) compounds from an aqueous solution. The results are shown in Table 2.

Thus, a simple sol–gel method for the synthesis of low-dimensional cobalt (II) ferrite has been applied. This method makes it possible to synthesize organo–inorganic composites in situ.

The results obtained are promising and could be used as a material synthesis method for a number of applications, such as targeted medication delivery.

## 3. Conclusions

A simple one-stage method for producing cobalt (II) ferrite has been proposed. The material structure formation mechanism is investigated, including the formation of a gel-like matrix with distributed transition elements cation inclusions in it. During gel thermal decomposition, nanosized ferrite particles are formed.

Using the method described, organo–inorganic composite materials based on cobalt (II) ferrite and an organic carrier are possible to obtain, as the study proved. Unlike existing analogues, the method allows to obtain composites in situ.

Composite materials formation is established to lead to a significant (5–9 times) increase in the surface area of the samples. Materials with a developed surface are formed: the surface area measured by the BET method is 83–143 m^2^/g.

The resulting composite materials have sufficient magnetic properties to be mobile in a magnetic field. Hence, wide possibilities open up for the synthesis of polyfunctional materials for various applications.

## 4. Materials and Methods

### 4.1. Materials

The following solutions were used as starting materials: cobalt (II) nitrate with the concentration of 183 g/L; iron (III) nitrate with the concentration of 242 g/L; an ammonia solution of 25% (wt.); and a 1200 g/L solution of citric acid. Analytical purity reagents of Co(NO_3_)_2_·6H_2_O, Fe(NO_3_)_3_·9H_2_O, NH_3_·H_2_O, C_6_H_8_O_7_ were used.

### 4.2. Synthesis of Biochar

To test an organic–inorganic composite material synthesis possibility, sunflower husks biochar and activated carbon from coconut shells were used as organic doping agents. The choice is determined by the availability of organic matter, its low cost, as well as the need to dispose of agricultural waste (sunflower husk and coconut peel) to obtain products with high added value. Sunflower husk biomass was produced in a muffle furnace by pyrolysis in a sealed metal vessel at the temperature of 100–700 °C with a temperature change step of 200 °C. The temperature rise rate was 11 °C/min. The holding time at temperatures of 100, 300, 500 °C was 20 min; at a temperature of 700 °C it was 45 min. Ready-made activated charcoal from coconut shells was used.

### 4.3. Material Synthesis

Cobalt (II) ferrite and composite materials synthesis was carried out in one stage with the formation of CoF_2_O_4_ and an in situ organic–inorganic composition. The formation of cobalt (II) ferrite on the surface of a carbon-containing substance was carried out according to the method developed by the authors and described in [17,18]. In a typical procedure, 25 mmol of Co(NO_3_)_2_ and 50 mmol of Fe(NO_3_)_3_ were simultaneously placed in a stainless-steel reaction vessel under continuous stirring. Then, 200 mmol of ammonia and 156 mmol of citric acid were introduced. The mixture was then heated until the liquid completely evaporated. Consequently, a viscous gel-like substance was formed, which gradually turned into a solid porous material. With further heating, a process of intense decomposition occurred, accompanied by the release of gaseous substances and the reaction system glowing. Heating continued until gaseous products of metal nitrates thermolysis stopped emitting. To obtain a composite organic–inorganic material, 25 g of biochar were first placed in the reaction vessel. Next, the synthesis procedure described above was carried out. The samples were designated as: FC for cobalt (II) ferrite, FC/S for cobalt (II) ferrite and sunflower husk biochar, FC/C for cobalt (II) ferrite and coconut shell activated carbon.

### 4.4. Characteristics

The following methods were used to characterize the resulting composite materials: X-ray diffraction (XRD), transmission electron microscopy, Brunauer–Emmett–Teller (BET) analysis, and the Scherrer method.

The phase composition was studied on an ARL X’TRA X-ray diffractometer (Ecublens, Switzerland) (monochromatized Cu-Kα radiation used) by point-by-point scanning (step of 0.01°, accumulation time at a point of 2 s) in the range of 2θ values from 20 °C to 70 °C. The crystallite size was calculated along line 311 using the Scherrer Equation (5):

*D* = 0.94·λ/(*B*·cosθ) (5)

where *D* is the average crystal size, nm, λ is the wavelength of X–ray radiation, nm, *B* is the value of the peak line width at half its height, rad., cosθ is the cosine value of the angle for the peak.

The calculation was carried by peak 311 (designated D_311_) and 400 (designated D_400_), the middle value (D_m_).

The surface area was determined on a ChemiSorb 2750 V apparatus. Nitrogen physical adsorption isotherms were obtained at 77 °K. Prior to measurement, the samples were degassed.

Ultrastructural images of the samples were obtained on a Tecnai G12 BioTwins transmission electron microscope (FEI, Philips, Czech Republic, Černovice) in bright field mode at an accelerating voltage of 100 kV.

The magnetic characteristics were measured using a Lake Shore VSM 7404 model magnetometer at the Shared Use Center of the International Research Institute for Intelligent Materials of the Southern Federal University, Russia, at room temperature (300 °K) with a maximum field value of 18 kOe. The test sample was fixed on a quartz rod, placed in an electromagnetic field. The rod with the sample was driven by a mechanical oscillator. As a result, the saturation magnetization M_S_ of the obtained samples was determined.

The adsorption activity of the synthesized materials was studied on a model solution of potassium dichromate with a concentration of 5 mmol/L. In this case, 5 mL of the initial potassium dichromate solution was passed through a reaction column containing 2 cm^3^ of the adsorbent. Next, 5 mL of deionized water was passed through the column, and the content of dichromate ions in the washing solution was determined. The determination was carried out by the intrinsic color of the solution by the photocolorimetric method using a KFK-2-UHL 4.2 device (Yurga, Russia) with a wavelength of 364 nm. The degree of purification (N) was calculated by Equation (6):

*N* = *m*_d_·/*m*_s_ (6)

where *N* is the adsorption capacity of the sample, mg/g, *m*_d_ is the mass of desorbed Cr_2_O_7_^2–^ ions, *m*_s_ is the mass of the sample, g.

## Figures and Tables

**Table 1:** Synthesized materials characteristics.

Sample	*a*, nm	*D*_311_, nm	*D*_400_, nm	*D*_m_, nm	*S*_BET_, m^2^·g^−1^
FC	0.8386	11.6	8.8	10.2	16
FC/S	0.8386	12.1	5.3	8.7	83
FC/C	0.8381	9.9	8.8	9.4	143

**Table 2:** Adsorption properties of synthesized materials.

Sample	*m*_s_, g	*m*_d_, mg	*N*, mg·g^−1^
FC	0.020	0.0008	0.04
FC/S	0.026	0.1815	6.98
FC/C	0.023	0.0161	0.70

## Data Availability

Not applicable.
